# ATEdrug: A reliable human-in-the-loop annotation scheme for aspect term extraction and polarity detection in drug reviews

**DOI:** 10.1371/journal.pone.0344296

**Published:** 2026-04-09

**Authors:** Gunjan Ansari, Chandni Saxena, Shahab Saquib Sohail, Zain Hussain, Aziz Sheikh, Amir Hussain

**Affiliations:** 1 VIT Bhopal University, Sehore, Madhya Pradesh, India; 2 The Chinese University of Hong Kong, Hong Kong SAR, China; 3 Department of CSE, SEST, Jamia Hamdard, New Delhi, India; 4 Edinburgh Medical School, Univesity of Edinburgh, Edinburgh, United Kingdom; 5 College of Medical, Veterinary and Life Sciences, University of Glasgow, Glasgow, United Kingdom; 6 Nuffield Department of Primary Care Health Sciences, University of Oxford, Oxford, United Kingdom; 7 Centre of AI and Robotics, Edinburgh Napier University, Edinburgh, United Kingdom; Philadelphia University, JORDAN

## Abstract

Pharmacovigilance is vital for post-market drug safety monitoring. Traditional trials inadequately capture adverse reactions. Patient-generated opinions offer valuable insights but pose challenges due to limited availability, inefficient annotation scheme, and volume of drug reviews, indicating the need of automation. To address the challenge posed by the limited availability of annotated drug reviews, we introduce a novel mechanism of annotating drug reviews dataset through human intervention, specifically tailored for Aspect Term Extraction (ATE) and Polarity Detection (PD) in three medical conditions: Depression, Arthritis and Birth control, thereby making it publicly available to facilitate future research. We first designed, ATEdrug, an automated annotation scheme with minimal human intervention by employing an expert-driven rule-based approach. We further deploy ATEdrug for sequence labelling and classification tasks in drug reviews. The automatically labeled data is further used for training and testing of deep learning models: BERT, BioBERT, and ClinicalBERT, for clinical use. We manually evaluate the results through annotator agreement to validate the effectiveness of ATEdrug. The labeled dataset is further used to construct transformer-based models. Our proposed model is reliable, safe and trustworthy for healthcare domain, thereby eliminating the compromises with hallucination and data security through generative AI models. All experimental codes and data are available on GitHub (https://github.com/CMOONCS/ateDrug.git).

## Introduction

The burgeoning field of pharmacovigilance underscores the necessity of monitoring drug safety post-market, a task significantly complicated by the limitations inherent in traditional clinical trials [1]. These trials, often constrained by selective patient demographics and treatment conditions, fail to comprehensively capture the spectrum of potential adverse drug reactions (ADRs).

Moreover, in healthcare sector, patient-generated opinions regarding their experiences with pharmaceutical products are of paramount importance, providing crucial insights for both pharmaceutical companies and potential consumers. Patients often consult the experiences of others regarding specific medications to guide their healthcare choices, while pharmaceutical companies use this feedback to assess and improve the effectiveness of drugs and safety. Nevertheless, the volume and unstructured nature of online patient feedback present significant challenges. The vast array of comments distributed across the web, primarily in textual and unstructured formats, renders traditional manual analysis methods both impractical and inefficient due to their time-consuming nature. Weblogs, discussion forums, user review websites, and social networking sites (e.g., Facebook and Twitter) are commonly used to express opinions about various subjects including drug reviews. Such data sources are widely available on internet where drug users and caregivers are expressing their opinion about medications in the form of text, reviews, posts, and comments. In response to these challenges, the field of sentiment analysis using machine learning [2,3] and deep learning architectures [4] on online drug reviews has emerged as a vital area of research.

Sentiment analysis or opinion mining is categorized into three analytic levels: document, sentence, and aspect, each with distinct utility [5]. Aspect-Based Sentiment Analysis (ABSA) represents the most intricate level, targeting specific attributes of an entity within user opinions, distilling insights from drug reviews on varied attributes like efficacy and side effects. Consider the following drug review P:

P: Contrave combines drugs that were used for alcohol, smoking, and opioid cessation. People lose weight on it because it also helps control over-eating. I have no doubt that most obesity is caused from sugar/carb addiction, which is just as powerful as any drug. I have been taking it for five days, and the good news is, it seems to go to work immediately.

In a given review P, the aspect term extracted is *weight loss* and the polarity is *positive* as observed through the text, *good news is, it seems to go to work immediately*.

While the interest in ABSA for drug reviews is escalating, as evidenced by recent studies, the automated aspect extraction and classification remains a relatively nascent area [6]. Existing research underscores the potential of supervised machine learning and deep learning models in aspect categorization, albeit constrained by the need for extensive, manually labeled datasets, a process that is often resource-intensive. Past studies observed major challenges in automated aspect term extraction and sentiment analysis performance in the healthcare sector that fall short of the current state-of-the-art standards. The study [7] shows that it is even more complex to identify implicit aspect terms that lack explicit names to indicate their presence.

Motivated with the need of employing ABSA on drug reviews, we use online drug reviews to computationally transform this vast, unstructured dataset into quantifiable insights. We hypothesise that our automated approach shall promise a more detailed, context-sensitive understanding of drug performance and patient sentiment post-release, thereby informing safer pharmaceutical practices and enhancing Clinical Decision Support Systems (CDSS) effectiveness.

**Problem**: In this research, we design a framework to conduct aspect-based polarity detection of drug reviews through computationally-driven aspect detection, uncovering public opinion for pharmacovigilence. Our major contributions are:

- To address the challenge posed by the limited availability of annotated drug reviews, we introduce a novel annotation scheme with iterative human intervention driven by experts. ATEdrug offers an efficient solution for automatic annotation of drug reviews for sequence labelling (aspect term extraction) and classification (polarity detection) tasks.
- We construct an automatically annotated drug review dataset consisting of 16,940 samples, specifically related to Birth Control, Depression, and Rheumatoid Arthritis. We perform human evaluation on all three medical conditions to validate the robustness of ATEdrug.
- To further assess the effectiveness of the proposed methodology, we develop fine-tuned transformer-based models on the annotated dataset, thereby facilitating future research.

**What is already known**: Aspect based sentiment analysis for drug reviews offers deep insights into customer emotions and preferences for subsequent drugs.

**What this paper adds**: To handle the problem of limited availability of dataset due to manual annotations, we propose rule-based computationally intelligent approach, ATEdrug, as human-in-the-loop annotation scheme for ATE and PD in drug reviews.

## Related work

In our exploration of advanced sentiment analysis techniques, we draw inspiration from DLIREC [8], an aspect term extraction and term polarity classification System which adeptly combines Conditional Random Fields (CRFs) and linear classifiers for Aspect-Based Sentiment Analysis [7,9]. This innovative integration of lexicon, syntactic, semantic, and cluster features has set a benchmark in the field, achieving unparalleled accuracy in aspect term extraction across diverse domains [10,11].

A recent study in sentiment analysis for health and well-being [12] shows that opinions shared by patients on medicines and healthcare services can help in their improvement, thus affecting the health outcomes. The authors emphasized the necessity for researchers to develop a vast, anonymized dataset that would serve as a benchmark for investigating current learning based methods.

Earlier, the research community used rule-based approaches to annotate healthcare dataset for Sentiment analysis, establishing the manual feature engineering through NLP tools. One of the most challenging tasks is identification of implicit and explicit aspect terms from the unstructured text due to limited manual domain knowledge [13]. As they construct dataset, they have enhanced their dictionary through external sources to map aspect terms in drug reviews. Furthermore, the research community used dependency relations [14,15], transfer learning [16], and graph-based Convolutional network [17] to obtain valuable insights from pharmaceutical review sites for improving health monitoring systems.

To handle the issue of data scarcity and extraction of implicit terms in the domain of drug reviews, the authors [18] proposed a novel multi-task learning that employed dual Bidirectional LSTM models for Aspect based Sentiment Analysis.

A recent survey on Aspect-based Sentiment Analysis [19,20] reports two major observations: (i) there still remains a challenge in developing automated annotation scheme for healthcare datasets; (ii) the selection of appropriate word embedding is essential to improve the performance of deep learning models.

Past studies have used domain-specific knowledge with deep learning models [21], rule-based approach with deep Convolutional Neural Network [22], and deep learning architectures [4] for sentiment analysis in the pharmaceutical domain.

Motivated by the use of review sites and drug domain-specific knowledge base to automate the construction of training set [13], we employ domain-specific seed words related to drug side-effects, obtained through external sources. The authors used distant supervision approach to generate aspect-tagged review sentences and their approach could achieve maximum F1-score of 78.05% using BERT model for aspect term classification. Another automatic annotation approach for drug reviews [17] used Graph Convolution Network for classification with dependency parse tree of review sentences. The results indicated in their study shows that the proposed approach gives better performance as compared to LSTM and RNN and could achieve a F1-score of 81.79%.

However, we further improve the rule-based aspect term extraction by infusing biomedical tagging and dictionary, and knowledge enhancement through additional external resources. Finally, we design an human-in-the-loop annotation scheme, ATEdrug, for aspect-term extraction and polarity detection. As compared to the existing works, our proposed approach is validated with human intervention for both aspect term and polarity classification. This ensures the robustness of the proposed work in identifying the aspect terms in the healthcare domain.

## Proposed method

**Fig 1:**
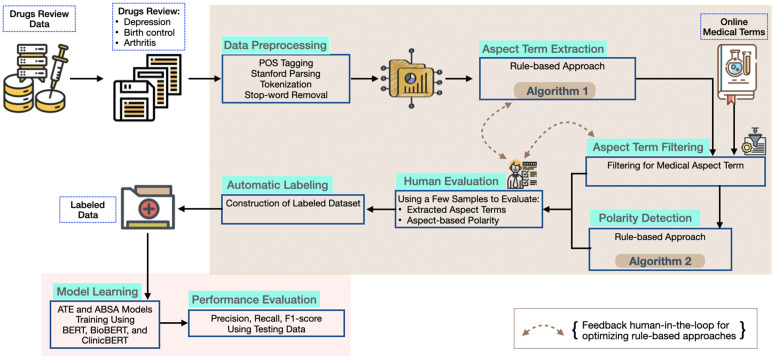
The overview of ATE_DRUG_ framework.

The proposed ATEdrug framework shown in Fig 1 aims to enable domain-specific information extraction from drug reviews, which are acquired for different diseases. It specifically focuses on aspect-term extraction and polarity analysis, allowing for a comprehensive understanding of the various aspects and sentiments expressed in the reviews. We annotate polarity for the extracted aspect terms as: (i) positive, (ii) negative, or (iii) neutral, to examine the patient-generated opinions. To resolve the limitation of the available annotated dataset, we collect the Kaggle dataset and construct an annotation scheme using a human-in-the-loop automated mechanism through a rule-based approach. In addition, we make this dataset publicly available to facilitate future research.

### Data collection

**Fig 2:**
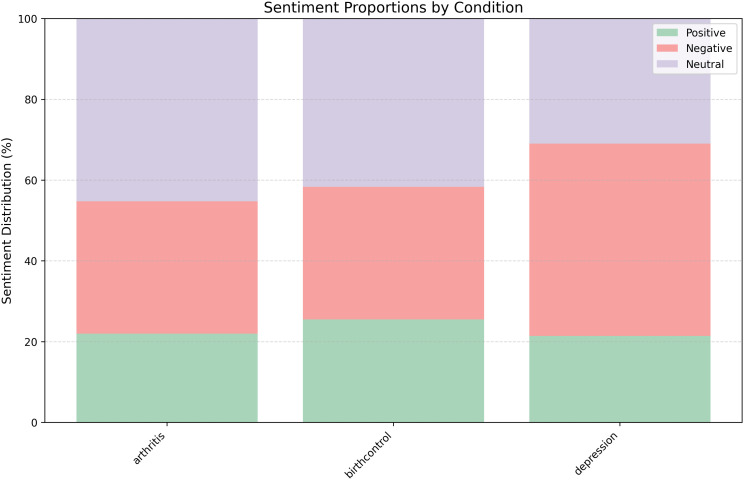
Distribution of sentiment proportion by disease conditions.

As we observe limited availability of the data set in the existing literature, we collected a data set of drug reviews from Kaggle [23] that consists of 161297 training samples considering the name, condition and review text of the drug. We filter reviews based on the 885 different conditions and then select drug reviews on three medical conditions, based on the availability of a sufficient number of samples in the data set. We use these human-generated drug reviews for three medical conditions: *Birth Control, Depression and Rheumatoid Arthritis* for our research. The characteristics of the collection of the data sets are given in Table 1. The distribution of the aspect phrases with their sentiment classes is provided in Table 2 and a portion of the sentiment distribution for the three different classes-positive,negative, and neutral for the selected conditions is shown in Fig 2.

**Table 1:** Dataset Description.

Condition	#Samples	Samples selected	Examples
Birth Control	28788	6921	“This medicine is absolutely terrible. After three months of using it my hair has fallen out so much so that I can see my scalp very visibly and its very very embarrassing. I stopped taking it and am now considering a copper iud. No more hormones for me.”
Depression	9070	9070	“1 week on Zoloft for anxiety and mood swings. I take 50mg in the mornings with my breakfast. Nausea on day one but that subsided as the week went on. I get the jitters about 2 hrs after taking it followed by yawning. I feel much better though and less angry/stressed.”
Rheumatoid Arthritis	995	995	“I was diagnosed a month ago with rheumatoid arthritis. My doctor started me on Plaquenil 2 weeks ago. 300 mg 2x a day. I had to stop taking it after a week because it upset my stomach causing vomiting, nausea, and extreme diarrhea. My doctor tried lowering my dose to 150 mg 1x a day to no avail. I m hoping that she can put me on something that will work better as I have been in extreme pain for over a year. I am 30 years old.”

**Table 2:** Aspect phrase with sentiment class.

Condition	Aspect Phrase	Positive	Negative	NC
	Count	Count	Count	Count
Depression	19833	4243	9442	6148
Birth Control	25961	6622	8512	10827
Arthritis	1736	381	569	786

### Preprocessing

Our data set, which comprises drug reviews, is particularly challenging due to its noise, a mixture of superfluous information that can impede text analysis, such as irrelevant spaces, numbers, and stop words. To enhance the efficiency and accuracy of our text analysis, we initiate preprocessing by removing these extraneous elements and converting all text to lowercase, ensuring uniformity and reducing complexity. Following this initial cleanup, we segment the text into individual sentences and further break these down into tokens, a process that allows us to analyze the data at a granular level. We then utilize ScispaCy’s POS tagger to annotate each token with its grammatical role.

### Aspect term extraction

The aspect term extraction algorithm is designed as a structured, rule-based method for parsing and identifying key terms that indicate aspects of drug reviews. Our rule-based method primarily focuses on the linguistic properties of the word, leveraging the Parts-of-Speech (POS) tags and dependency relations to identify relevant terms.

From the open-source library ScispaCy, we first employ the specialized spaCy models (en_core_sci_lg model) that are tailored to preprocess the biomedical and clinical datasets. The en_core_sci_lg model includes a comprehensive spaCy pipeline, equipped with a tokenizer, a parser, and a POS tagger, all of which are essential for NLP tasks. The model boasts a substantial vocabulary that covers a wide array of biomedical terms, which is crucial for domain-specific analysis. Additionally, it contains 600,000 word vectors, trained on biomedical text, and thus providing us with the nuanced semantic representation needed for accurate NLP operations.

**Fig 3:**
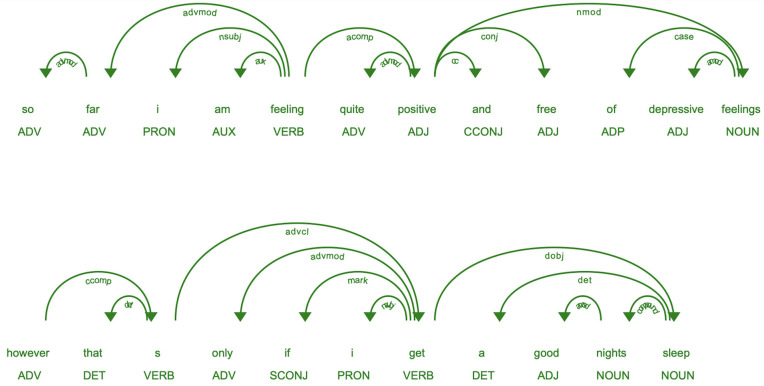
Screenshot of Sample 1 and 2.

In addition to POS tagging, we applied ScispaCy’s dependency parser to establish the syntactic dependencies between tokens. This parsing is pivotal in discerning the relationships and hierarchical structure within sentences, which is particularly beneficial for subsequent tasks that require an in-depth understanding of the semantics. The Fig 3 shows the result of the dependency parser and POS tagging using ScispaCy on the sample review sentences R1 and R2 from the drug reviews as shown below:

**R1** = “ so far im feeling quite positive and free of depressive feelings ”

**R2** = “ however that’s only if we get a good nights sleep ”

As observed from this review sentence, NOUN, ADJECTIVES and ADVERB play a significant role in aspect term extraction and polarity detection.

**Algorithm 1 Rule-Based Aspect Term Extraction**

**Require:** sentence (a list of tokens with POS and DEP tags)

**Ensure:** RevisedAspectTerms (a filtered list of aspect terms)

1: Initialize FLAG ← 0

2: Initialize PREPEND ← empty string

3: Initialize TAG ← empty string

4: Initialize AspectTerms ← empty list

5: Initialize RevisedAspectTerms ← empty list

6: **for** each token in sentence **do**

7:   **if** POS(token) = NOUN and PREPEND = empty and (DEP(token) = ROOT or DEP(token) = nmod) **then**

8:    Append token to AspectTerms

9:   **else if** POS(token) = NOUN and DEP(token) = compound **then**

10:    PREPEND ← token

11:    TAG ← “comp”

12:   **else if** PREPEND ≠ empty and TAG = “comp” **then**

13:    Append PREPEND + “ ” + token to AspectTerms

14:    PREPEND ← empty

15:   **end if**

16:   **if** POS(token) = ADJ and DEP(token) = amod **then**

17:    FLAG ← 1

18:   **else if** POS(token) = NOUN and FLAG = 1 **then**

19:    Append token to AspectTerms

20:    FLAG ← 0

21:   **end if**

22: **end for**

23: **for** each aspectTerm in AspectTerms **do**

24:   sim ← ComputeSimilarity(aspectTerm, mdict)

25:   **if** sim ≥ 0.7 **then**

26:    Append aspectTerm to RevisedAspectTerms

27:   **end if**

28: **end for**

29: **return** RevisedAspectTerms

**Algorithm 2 Sentiment Polarity Detection**

**Require:** A review sentence *R*, list of aspect terms *ASPECT*, modifier lists *STRONG*, *WEAK*, *NEGATIVES*

**Ensure:** *polarity* score

1: Initialize FLAG ← 0, polarity ← 0

2: **for** each token in *R* **do**

3:   **if** token ∈ *ASPECT* **then**

4:    **continue**

5:   **else if** modifier(token) = TRUE **then**

6:    **if** token ∈ *STRONG* **then**

7:     FLAG ← 1

8:    **else if** token ∈ *WEAK* **then**

9:     FLAG ← 2

10:    **else if** token ∈ *NEGATIVES* **then**

11:     FLAG ← 3

12:    **end if**

13:   **else if** sentiment(token) = TRUE **then**

14:    (pos,neg,obj)← getPolarity(token)

15:    **if** obj≥Θ **then**

16:     **continue**

17:    **else if** pos≥neg **then**

18:     pos←1, polarity←pos

19:    **else**

20:     pos←0, polarity←1−neg

21:    **end if**

22:    **if** (FLAG=1∧pos=1) or (FLAG=2∧pos=0) **then**

23:     $polarity\leftarrow 1-(1-polarity{)}^{2}$

24:    **else if** (FLAG=1∧pos=0) or (FLAG=2∧pos=1) **then**

25:     $polarity\leftarrow 1-\sqrt{\left(1-polarity\right)}$

26:    **else if** (*FLAG* = 3) and (*pos* = 0 or *pos* = 1) **then**

27:     polarity←1−polarity

28:    **end if**

29:   **end if**

30: **end for**

31: **return** *polarity*

Algorithm 1 has a systematic process of identifying and categorizing relevant tokens within sentences. Initially, key variables are established, including flags for tracking conditions and variables to manage compound nouns. The algorithm iterates over each word in the sentence, distinguishing nouns as either sentence roots or modified forms. Compound nouns are identified and processed accordingly, with compound components concatenated for inclusion in the aspect terms list.

Moreover, the algorithm identifies adjectives modifying nouns, signifying potential aspect terms. This involves setting flags and verifying noun adjacency, allowing for the extraction of pertinent terms. Our approach is exemplified through sample sentences, illustrating how the algorithm accurately identifies multi-word aspect terms and effectively handles adjective-noun combinations. Thus, our algorithm offers a systematic and efficient methodology for aspect term extraction, facilitating nuanced analysis of textual data. Its robustness is demonstrated through its ability to accurately capture complex linguistic structures and extract relevant terms, thereby enhancing the comprehensiveness and precision of text analysis tasks.

### Aspect term filtering

We developed a filtering methodology to refine the extraction of aspect terms initially captured using a rule-based approach, which often includes few irrelevant terms along with aspect tokens. Our goal was to identify and keep only those terms that are closely related to the specific medical condition being studied by leveraging advanced semantic processing techniques. To accomplish this, we compared each term from the initially extracted aspect list with terms from a medical vocabulary corpus, which we have compiled by scraping various online medical resources for Depression (https://www.everydayhealth.com/depression/speaking-depression-a-glossary-of-terms-used-to-describe-the-disorder/), Arthiritis (https://www.everydayhealth.com/rheumatoid-arthritis/guide/) and Birth Control (https://www.mayoclinic.org/tests-procedures/combination-birth-control-pills/about/pac-20385282). For the semantic processing, we utilized the ScispaCy pipeline en_core_sci_md, which is specifically tailored for biomedical text. This pipeline includes an extensive vocabulary and 50,000 word vectors that are optimized for biomedical data, allowing for effective vector representation of the terms.

We then calculated the cosine similarity between these vector representations using the similarity method provided in the pipeline. Based on a predefined threshold for similarity scores, we filtered the terms. Those exceeding the threshold were retained in the filtered aspect term list, ensuring they had substantial semantic relevance to the medical condition, while the rest were discarded. This approach allowed us to produce a more curated and accurate list of aspect terms. The final list is expected to enhance the reliability and precision of our analysis, focusing on aspects with significant semantic ties to the condition being studied. The details of this process and its effectiveness are further illustrated in Algorithm 1.

### Polarity detection

We further designed Algorithm 2 to assign polarity to the extracted aspect terms. We define a list of tokens as modifiers. We find POS tags and mark the tokens as opinion words or modifiers, based on a predefined set of rules.

We further assign positive, negative and objective (obj) scores to the opinion words in the review sentence using SentiWordNet [24]. If the obj score is greater than a threshold value, then the token carries no opinion so polarity remains zero. We obtain polarity (P) as


$$\left\{ \begin{array}{c} P>\theta 1⟹\mathrm{positiveP}\\ \theta 1\leq P\leq \theta 2⟹\mathrm{neutralP}\\ P<\theta 2⟹\mathrm{negativeP} \end{array}\right.$$ (1)

*Working Instance*: For instance, in one of the review sentence *I have been truly lucky as I have not experienced any side affects in the years I have been on Actemra*, the intensifier “truly” and the adjective “lucky” are associated with the aspect term “side effects,” resulting in a positive sentiment score for this term. This score is further enhanced by the use of the modifier in the sentence. Despite the presence of the negation word “not,” its influence does not negate the sentiment, as it modifies the neutral sentiment word “experience,” leaving the positive sentiment intact.

In contrast, the analysis of another review *so far im feeling quite positive and free of depressive feelings* reveals the use of “quite” as a weak modifier and “positive” as an adjective linked to the aspect term “feelings”. Here, the weak modifier dilutes the sentiment strength, thereby assigning a neutral polarity to the term “feelings” instead of a positive one, demonstrating the impact of modifiers on sentiment scoring in aspect-based sentiment analysis.

### Our approach

In this research paper, we first acquire a dataset of drug reviews from Kaggle, laying the foundation for our investigation. We then assemble a panel of experts to craft rules guiding the identification of aspect terms and their associated polarity within these reviews. Our approach extends beyond conventional methods by integrating semantic analysis techniques and leveraging domain-specific resources such as dictionaries for biomedical terms related to the three drugs review areas. Through this innovative framework, ATEdrug, we introduce a novel human-in-the-loop annotation scheme to facilitate the information extraction from user-generated opinions on drug usage, offering a robust and reliable mechanism for annotating aspect-based sentiment analysis in the pharmaceutical domain.

We first utilize ATEdrug to identify aspect terms and determine their polarity. Subsequently, we conduct manual annotation on randomly selected samples from the generated dataset. In this annotation process, we employ human annotators and perform an agreement study to establish the ground truth for selected samples. Following this, we assess the performance of ATEdrug in both sequence labeling and classification tasks using performance evaluation measures. Upon finding substantial agreement and reliable outcomes across these tasks, we utilize the annotated dataset as our ground truth. This dataset will serve as a foundation for the development of language models tailored for healthcare applications in the future.

## Experimental setup and results

In our experiments and evaluation section, we employ a human-in-the-loop rule-based annotation scheme to meticulously evaluate the performance of two algorithms, namely Algorithm 1 and Algorithm 2, in addressing the critical tasks of ATE and PD within the domain of drug reviews. This annotation approach ensures a thorough examination of the algorithms’ efficacy by incorporating human expertise and domain knowledge. We extend our evaluation by utilizing transformer models to tackle the challenging learning tasks associated with extraction of aspect terms and their polarity detection using automatically annotated datasets.

This section offers an in-depth exploration of our experimental setup, encompassing dataset selection, configuration parameters, and the annotation methodology. Additionally, we introduce baseline transformers to serve as benchmarks for future dataset annotation through initial screening with human-in-the-loop annotation scheme. We then present the experimental findings, inclusive of precision, recall, and F1-score metrics, providing quantitative insights into the algorithms’ proficiency in aspect term identification and polarity determination. Furthermore, a qualitative analysis is conducted to elucidate the strengths and limitations of each algorithm in real-world scenarios.

Through this comprehensive assessment, we aim to illuminate the capabilities of both traditional rule-based methods and modern transformer models in addressing the intricate tasks of ATE and PD within drug review contexts. This analysis not only sheds light on the practical implications of these algorithms but also steers future research endeavours in the domains of healthcare informatics and natural language processing.

### Experimental setup

For our experimental setup, we acquired a raw dataset from Kaggle, a popular platform for datasets and data science competitions. The dataset obtained from Kaggle serves as the foundation for our analysis and experimentation. To conduct our analysis and experiments, we use Python, a versatile programming language widely used in NLP tasks. We used Python version 3.7 for compatibility with the essential libraries such as NLTK (Natural Language Toolkit), spaCy, and Transformers. For rule-based methods, we utilized libraries such as pandas for data manipulation and scikit-learn for implementing machine learning algorithms.

In terms of hardware requirements, our experiments were conducted on a standard workstation with 16GB of RAM and a multi-core processor to ensure smooth execution of experiments involving large datasets and complex models. These tools and resources enable us to conduct comprehensive analyses and evaluations of aspect term extraction and polarity detection techniques within the context of drug reviews.

### Ground truth: Manual annotation

In our research, we engaged three human annotators to meticulously annotate selected samples, with 300 samples chosen from each dataset. These annotations were conducted following predefined rules and an annotation scheme guided by domain expertise. The annotation tasks encompassed two main objectives: (i) sequence labeling for aspect term extraction and (ii) classification tasks for polarity detection of the extracted aspect terms. To ensure the accuracy and reliability of the annotations, we established a ground truth dataset based on a voting mechanism, where the consensus among annotators determined the final label for each sample, aiding mitigate individual biases and discrepancies.

To evaluate the agreement between annotators, we employed Cohen’s Kappa (κ), a statistical metric widely used for assessing the degree of agreement in classification tasks. For sequence labeling, κ was used as the agreement study mechanism, resulting in an overall agreement of 78.34%. In general, scores above 60.0% are considered acceptable, with 75.0% or higher indicating excellent reliability. These results show consistency and reliability of aspect term extraction by human annotators.

Furthermore, for classification tasks such as polarity detection, we utilized Fleiss’ Kappa to assess inter-rater agreement. In our study, Fleiss’ Kappa yielded a value of 75.91%, indicating substantial agreement among annotators in determining the polarity of extracted aspect terms. The high values obtained for both Cohen’s Kappa and Fleiss’ Kappa demonstrate significant agreement among annotators and validate the robustness of our annotation process. This level of inter-annotator agreement is deemed acceptable for further analysis and ensures the reliability of our annotated dataset.

### Performance evaluation: ATEdrug

We deploy ATEdrug to analyze drug reviews related to three medical conditions and assess Aspect Term Extraction (ATE) and Polarity Detection (PD) tasks. From our dataset, we randomly select 300 samples for each medical condition and evaluate ATEdrug’s performance using ground truth data.

For aspect term extraction, we compare the aspect terms identified by human annotators, termed as ‘Actual’ tokens, with those annotated by ATEdrug, termed as ‘Predicted’ tokens. The overlap between ‘Actual’ and ‘Predicted’ terms is considered ‘Correct’ tokens. We compute precision, recall, and F1, using the formula shown in equation below:


$$Precision=\frac{Correct}{Predicted}$$ (2)

$$Recall=\frac{Correct}{Actual}$$ (3)

$$F1=\frac{2*Precision*Recall}{Precision+Recall}$$ (4)

The results of evaluation are detailed in Table 3. We continuously refine our expertise-driven rules to ensure the creation of significantly annotated data. Our experimental findings demonstrate improved results, with F1-scores exceeding 80%, utilizing a human-in-the-loop annotation scheme. This iterative process underscores the importance of expert guidance in enhancing the accuracy and reliability of our results.

**Table 3:** Performance evaluation of human-in-the-loop automated aspect term extraction.

Dataset	#Instances	#samples	Precision	Recall	F1-score
Depression	9069	300	79.41	**80.59**	80.00
Birth Control	6921	300	**87.98**	75.41	81.21
Arthiritis	950	300	86.17	77.33	**81.50**

For polarity detection, the drug review samples are categorized as 0 for Negative, 1 for Neutral, and 2 for Positive sentiments. We employ performance evaluation measures to assess the effectiveness of the ATEdrug polarity detection mechanism across various medical conditions (see Table 4). Thus, ATEdrug demonstrates robust performance in both aspect term extraction and polarity detection across diverse medical conditions.

**Table 4:** Performance evaluation of human-in-the-loop automated polarity detection.

Class	Depression Dataset			Arthritis Dataset			Birth Control Dataset		
	P	R	F1	P	R	F1	P	R	F1
Negative	**0.85**	0.76	0.80	**0.92**	0.80	**0.85**	0.65	**0.79**	0.71
Neutral	0.75	**0.91**	**0.82**	0.91	0.65	0.72	**0.96**	0.72	**0.82**
Positive	0.73	0.75	0.74	0.51	**0.86**	0.64	0.75	0.77	0.76

### ATEdrug enhanced transformer models

In our study, we implement a human in-the-loop annotation scheme, incorporating iterative human intervention to establish the ground truth of our dataset. This approach ensures meticulous annotation, leveraging human expertise to refine and validate the dataset annotations. Furthermore, we utilize ATEdrug alongside transformer-based models for both sequence labeling, pertaining to Aspect-Term Extraction (ATE), and classification tasks, related to Polarity Detection (PD). In this section, we delve into the details of the baseline methods and hyperparameters employed for the transformer-based models utilized in our work. By providing this detailed exposition, we aim to offer transparency and reproducibility in our experimental setup and methodology.

#### Baselines.

We employed the BERT-based model [25] along with fine-tuned ClinicalBERT [26] and BioBERT [27] models. The reason for choosing these three models for training and testing is due to their pre-training data and fine-tuning strategies, making them valuable for sequence labelling and classification tasks across medical domains.

#### Runtime configurations.

In the proposed method, an extra output layer is added to the model to customize it for the specific task of aspect-term extraction. The aspect term extraction that employed sequence labeling is built to determine whether a given token is an aspect or not.

The classification model undergoes fine-tuning, primarily focusing on assigning sentiment labels (positive, negative, neutral) to the input text with associated aspects. Aspect Term Extraction (ATE) and Polarity Detection (PD) models are trained with the following hyperparameter settings: a learning rate of $2\times 10^{-5}$, a training batch size of 10, a testing batch size of 50, and 3 epochs. These parameter selections are made to facilitate efficient training and to achieve the desired outcomes.

To assess the performance of the fine-tuned models on the ATE task, tokens from the testing samples are categorized into three classes: Non-aspect tokens, Uni-word aspect tokens, and bi-word aspect tokens. Performance metrics such as precision, recall, and F1-score are computed using correctly identified tokens across all three classes.

#### Experimental results.

Table 5 provides detailed performance metrics for three models—BERT, BioBERT, and ClinicalBERT—across two tasks.

**Table 5:** Performance Analysis of Deep Learning Models for Aspect Term Extraction and Polarity Detection.

Model	BERT			BioBERT			ClinicalBERT		
	P	R	F1	P	R	F1	P	R	F1
**Aspect Term Extraction**									
Non-Aspect	**0.98**	**0.96**	**0.97**	**0.96**	**0.97**	**0.96**	**0.98**	**0.94**	**0.96**
Uniword	0.89	0.86	0.87	0.87	0.88	0.88	0.86	0.71	0.76
Bi-word	0.84	0.81	0.82	0.85	0.79	0.82	0.79	0.66	0.72
**Polarity Detection**									
Negative	**0.91**	0.87	**0.89**	0.86	0.91	**0.88**	**0.98**	0.83	0.86
Neutral	0.80	0.84	0.82	**0.96**	0.64	0.76	0.90	0.66	0.77
Positive	0.89	**0.90**	**0.89**	0.82	**0.94**	**0.88**	0.86	**0.90**	**0.87**

**Aspect Term Extraction**: For class 0 (Non-aspect Tokens), all models demonstrate high performance in identifying non-aspect tokens, with BERT and BioBERT achieving the highest F1-scores of 0.97, and ClinicalBERT slightly lower at 0.96. This indicates a strong capability in distinguishing non-aspect terms across the models. For class 1 (Uniword Aspect Terms), BERT shows the highest F1-score of 0.87, suggesting it is better at identifying single-word aspect terms compared to BioBERT (0.88) and ClinicalBERT (0.76), the latter showing notably weaker performance. For class 2 (Biword Aspect Terms), BERT and BioBERT are relatively close, with F1-scores of 0.82 and 0.82, respectively. ClinicalBERT falls behind significantly at 0.72, indicating that it struggles more with bi-word aspect term identification.

**Polarity Detection**: For negative sentiments, BERT performs best with an F1-score of 0.89, followed by BioBERT at 0.88 and ClinicalBERT at 0.86. This shows that BERT slightly outperforms the others in identifying negative sentiment. For neutral sentiments: BioBERT leads in identifying neutral sentiments with an F1-score of 0.76, despite a lower precision. BERT has a lower F1-score of 0.82 despite higher precision and recall, indicating a disparity in the balance of its metrics. ClinicalBERT shows the lowest performance at 0.77. For positive sentiments, BERT and ClinicalBERT are competitive in detecting positive sentiments, with F1-scores of 0.89 and 0.87, respectively. BioBERT, while close, is slightly lower at 0.88.

The differing performances of these models are influenced by their pre-trained domain knowledge. BERT generally exhibits the best performance across most categories, especially in the more complex task of polarity detection. BioBERT performs robustly in neutral polarity detection and is competitive in other areas. ClinicalBERT, while generally effective, shows certain weaknesses, particularly in identifying bi-word aspect terms and in some aspects of polarity detection.

### Are we ready to use Generative AI as an annotator for healthcare utilization?

With the advent of advanced language generation techniques, we utilized the Llama-2-7b model (https://huggingface.co/meta-llama/Llama-2-7b-chat) and fine-tuned Llama model on medical domain Medalpaca-7b (https://huggingface.co/medalpaca/medalpaca-7b) to evaluate the zero-shot adaptability of large language models (LLMs) on domain-specific biomedical user-generated reviews [28]. Our objective was to test and validate our hypothesis. Using 300 drug reviews, we performed zero-shot inference for ATE and PD tasks (see samples in Table 6). For the both models’ setup, we have used a temperature of 0.7, which strikes a balance between diversity and coherence in the generated outputs. The results, validated through human evaluation, indicate poor adaptability of LLMs on the domain-specific biomedical data. The prompt given to LLAMA model is as follows:

You are an expert in the biomedical field. You have a knowledge to extract aspect based terms and their sentiment polarity from the context of the drugs review sentences from the users. There are three sentiments polarities: positive, negative, or neutral for the aspect terms. Extract aspect terms and their polarity from the given review text.

**Table 6:** G: Generated aspect terms and their polarity using Llama-2-7B; GM: Generated aspects terms and their polarity using Medalpaca-7b T: True aspect terms with polarity by human annotator.

Input	G	GM	T
“I feel optimistic for the future, my sex drive is better. I have more energy amp am more focused. I have fibromyalgia as well so this is huge for me and I’ve quit smoking!”	sex drive -positive; fibromylgia -negative	improved sex drive -positive; increased energy-positive; improved focus- positive	sex drive -positive; energy -positive
“Like Celebrex and Vioxx this caused blood pressure spurts along with dizziness weakness and other side effects”.	side effects – negative	blood pressure spikes-negative	blood pressure -negative; weakness-negative; effects -negative
“Worried its a black box drug through! No side effects but maybe stomach feeling bloated”.	drug – neutral; bloated – positive	no side effects -neutral, stomach feeling bloated-neutral	side effects -negative; stomach -negative

The performance evaluation of the LLAMA model and its clinical variant - ‘Medalpaca-7b’ in the tasks of Aspect Term Extraction and Polarity Detection reveals several insights into its capabilities and limitations. In Aspect Term Extraction, LLAMA model exhibit moderate level of effectiveness achieving an F1-score of 0.60. However, ‘Medalpaca-7b’ model achieved a relatively lower f1-score of 0.45. Also, there is relatively high recall of 0.75 in case LLAMA, suggesting that the model is capable of identifying a significant portion of relevant aspect terms. In contrast, its variant achieves comparatively lower recall of 0.46. Further, the results indicate that both models captures a substantial amount of irrelevant data, as indicated by a lower precision of 0.50 and 0.43.

In Polarity Detection, the results are mixed across different sentiment classes. For negative sentiments, the LLAMA model and its clinical variant - ‘Medalpaca-7b’ performs reasonably well, with an F1-score of 0.66 and 0.76, supported by nearly balanced precision scores of 0.65 and 0.75 and recall scores of 0.65 and 0.78 respectively. This indicates a fairly reliable performance in identifying negative sentiments within the data. However, the models’ ability to detect neutral sentiments is notably poor, reflected by an F1-score of just 0.16 and 0.42, with precision of 0.15 and 0.58 and recall of 0.19 and 0.33. This suggests a significant challenge in accurately classifying neutral sentiments, where the model fails to identify the majority of relevant cases and frequently misclassified them. For positive sentiments, the performance is somewhat better than for neutral sentiments but still suboptimal, with an F1-score of 0.32 and 0.58 for LLAMA and its variant. The precision of 0.38 and a recall of 0.28 for LLAMA model indicate difficulties in consistently identifying positive sentiments accurately. However, ‘Medalpaca-7b’ shows a slightly better precision of 0.46 and much better recall of 0.75 over its base variant.

These findings highlight the strength of LLAMA and Medalpaca-7b in handling negative sentiments but underscore significant deficiencies in detecting neutral and positive sentiments especially in case of LLAMA base variant, as well as a general issue with precision in aspect term extraction for both variants.

In the healthcare utilization industry, there is an increasing demand for efficient and secure methods to analyze and annotate large volumes of healthcare data. As organizations strive to enhance their operational efficiency and safeguard patient privacy, it becomes imperative to critically evaluate the tools employed for such tasks. In this paper, we propose that instead of adopting the LLAMA model, a generative AI model, healthcare organizations should consider a human-in-the-loop, rule-based annotation scheme. This recommendation is based on several critical considerations: cost-effectiveness, accuracy, data security, and reliability. Considering these factors, a human-in-the-loop rule-based annotation scheme is not only a viable alternative but a preferable choice in the context of healthcare data annotation. [29]. It offers a balanced approach that leverages the accuracy and consistency of rule-based algorithms along with the critical oversight provided by human experts. This method ensures high-quality data processing while addressing the essential needs for cost efficiency, data security, and reliability, making it an ideal choice for healthcare organizations aiming to improve their data annotation practices.

## Conclusion

The transformer-based model can potentially extract informative data from opinions of drug users and use it for further improvements. The potential challenge in analyzing patient generated opinions for pharmaceutical companies, lies in the availability of the annotated dataset. The transformer-based model can potentially extract informative data from opinions of drug users that can be used for further improvements. We design and deploy expert-driven rule-based human-in-the-loop annotation schemes to obtain an annotated dataset.

The efficacy of ATEdrug is validated using a human evaluation method, resulting in a higher value of F1-score for both aspect term and polarity detection. The semantic technique used for aspect term filtering approach in the proposed work was able to achieve a high precision value of around 81%. The proposed approach is a cost-effective and efficient solution that can be used for automatic annotation of user opinion in the medical domain, specifically for drug reviews.

However, one of the limitations of the proposed rule-based approach for aspect term extraction is that it requires domain-specific terms for efficient working of aspect-term filtering. Also, determining an accurate threshold value for filtering using human intervention could pose a challenge for researchers in large datasets. The future work could use a hybrid of rule-based and deep learning approach to filter aspect terms. Further, explainability and interpretability of the proposed deep learning model can be enhanced by incorporating attention mechanism in the BERT model. The work can be extended for fine-tuning of Llama models to generate more accurate results. Also, comparative analysis of drugs for specific conditions based on the different aspects can be explored in the future. Future work can also focus on the detailed domain-specific analysis based on various deep learning models to complement this study.
